# An extended PROSPECT: Advance in the leaf optical properties model separating total chlorophylls into chlorophyll a and b

**DOI:** 10.1038/s41598-017-06694-y

**Published:** 2017-07-25

**Authors:** Yao Zhang, Jingfeng Huang, Fumin Wang, George Alan Blackburn, Hankui K. Zhang, Xiuzhen Wang, Chuanwen Wei, Kangyu Zhang, Chen Wei

**Affiliations:** 1 Institute of Applied Remote Sensing & Information Technology, Zhejiang University, Hangzhou, 310058 China; 2 Ministry of Education Key Laboratory of Environmental Remediation and Ecological Health, Zhejiang University, Hangzhou, 310058 China; 3 Key Laboratory of Agricultural Remote Sensing and Information System, Zhejiang Province, Hangzhou, 310058 China; 4 Lancaster Environment Centre, Lancaster University, Lancaster, LA1 4YQ UK; 5 Institute of Geospatial Science Center of Excellence, South Dakota State University, Brookings, SD 57007 USA; 6 Remote Sensing and Earth Sciences, Hangzhou Normal University, Hangzhou, 311121 China

## Abstract

The PROSPECT leaf optical model has, to date, well-separated the effects of total chlorophyll and carotenoids on leaf reflectance and transmittance in the 400–800 nm. Considering variations in chlorophyll a:b ratio with leaf age and physiological stress, a further separation of total plant-based chlorophylls into chlorophyll a and chlorophyll b is necessary for advanced monitoring of plant growth. In this study, we present an extended version of PROSPECT model (hereafter referred to as PROSPECT-MP) that can combine the effects of chlorophyll a, chlorophyll b and carotenoids on leaf directional hemispherical reflectance and transmittance (DHR and DHT) in the 400–800 nm. The LOPEX93 dataset was used to evaluate the capabilities of PROSPECT-MP for spectra modelling and pigment retrieval. The results show that PROSPECT-MP can both simultaneously retrieve leaf chlorophyll a and b, and also performs better than PROSPECT-5 in retrieving carotenoids concentrations. As for the simulation of DHR and DHT, the performances of PROSPECT-MP are similar to that of PROSPECT-5. This study demonstrates the potential of PROSPECT-MP for improving capabilities of remote sensing of leaf photosynthetic pigments (chlorophyll a, chlorophyll b and carotenoids) and for providing a framework for future refinements in the modelling of leaf optical properties.

## Introduction

Monitoring the biochemical constituents of plant leaves using remote sensing techniques can improve our understanding of the dynamics of vegetation physiological and ecological functions. In the 400–800 nm region, leaf photosynthetic pigments are the main absorbers of harvesting light in plants^1, 2^ and these pigments mainly consist of total chlorophylls (Chls) (chlorophyll a (Chla), chlorophyll b (Chlb)) and carotenoids (Cars)^3–5^. Chlorophyll a can act as a light harvesting pigment and the reaction centre for leaf photosynthesis^6^. Chlorophyll b can act as an accessory light-harvesting pigments and helps Chlorophyll a to perform leaf photosynthesis^7^. Carotenoids also act as accessory light-harvesting pigments and perform an essential photo-protective role on non-photochemical quenching of excess light energy^8^. As the physiological functions of these photosynthetic pigments in plant are different, their proportions vary according to leaf age and physiological stress. especially chlorophyll a and b. For instance, a decrease in chlorophyll a/b ratios in rice seedlings is associated with leaf senescence^9^, whereas an increase of the chlorophyll a/b ratios is associated with nitrogen limitation and high light during acclimation of tropical woody seedlings^10^. Therefore, an improved discrimination between the key pigments is important for physiological and ecological applications of remote sensing^11^. However, to date, efforts to simultaneously retrieve the concentrations of individual photosynthetic pigments from remotely-sensed data have been restricted due to the overlap of absorption spectra of different pigments which can mask the contribution of individual pigments to the reflectance and/or transmittance spectrum.

PROSPECT models are a type of leaf radiative transfer (RT) models, which have been widely used in the remote sensing community^4, 12^. The earlier versions of PROSPECT models only considered the effects of total chlorophylls pigments. Until PROSPECT-5 version, this model separated photosynthetic pigments into total chlorophylls and carotenoids. Within this version (PROSPECT-5), the separation of Chls and Cars absorption coefficients was band-by-band derived based on a minimum distance fitting method using modeled and measured *in vivo* spectra^13^. However, with this method, the specific absorption coefficients of Chla and Chlb could not be separated in PROSPECT-5^11^. This is because band-by-band fitting method is a purely mathematical fitting and absent of physical significance. Thus, uncertainties in indexing absorption peaks to their respective pigments may occur during the determination of these pigment absorption coefficients.

Therefore, a new approach with physical significance that can limit the masking phenomenon between multiple pigment spectra is needed in PROSPECT model. The Gauss-Lorentz (G-L) function fitting method can be used to define individual material absorption spectra and provide a chance to explicitly deal with the issue of overlapping absorption features^14–16^. G-L function fitting can be applied in the spectral absorption peak separation of mixed constituents by fitting the parameters (absorption peak height, full width at half maximum (FWHM) and Gauss ratio), given the absorption peak number and position^14^. These parameters holds explicit physical significance of material absorption spectra. As the given absorption peak positions can help the G-L function to index and characterize its own absorption spectra, the separation of absorption spectra of mixed materials is not susceptible to the problem of band overlapping and masking phenomena between absorption features^17^.

In view of the above therefore, the present study develops an algorithm for the separation of multiple photosynthetic pigment absorption coefficients (Chla, Chlb and Cars) by using a modified G-L function, and then proposes an extended version of the PROSPECT model in the 400–800 nm range, herein referred to as PROSPECT-Multiple Pigment (PROSPECT-MP). PROSPECT-MP is capable of incorporating the *in vivo* absorption coefficients of Chla, Chla and Cars pigments and describes leaf optical properties from 400 to 800 nm in order to facilitate the simultaneous retrieval of these multiple individual photosynthetic pigment concentrations by model inversion.

## Results and Discussion

### Parameter Calibration

#### Characteristics of the PROSPECT-MP parameter calibration

In order to describe the absorption features present within these spectra, we used the full width at half maximum (FWHM, ${{\boldsymbol{K}}}_{{\boldsymbol{i}},{\boldsymbol{j}},{\boldsymbol{w}}}$) and position of each absorption peak (${{\boldsymbol{K}}}_{{\boldsymbol{i}},{\boldsymbol{j}},{\boldsymbol{p}}}$) to derive the Range of Absorption Feature (${\boldsymbol{RAF}}$) using the following:

1a $$RA{F}_{i,j,lb}={K}_{i,j,p}-{K}_{i,j,w}/2;\,RA{F}_{i,j,ub}={K}_{i,j,p}+{K}_{i,j,w}/2.$$

Where

1b $${K}_{i,j,p}={A}_{i,j,p}+{K}_{i,j,{\rm{\Delta }}\lambda }$$

where *i* is the determinable pigment type (Chla, Chlb or Cars); ${j}$ is the peak number within the pigment-specific absorption coefficient;${A}_{{\rm{i}},{\rm{j}},{\rm{p}}}$ is the peak position of the ${\boldsymbol{j}}{\rm{t}}{\rm{h}}$ absorption peak for the ${\boldsymbol{i}}{\rm{t}}{\rm{h}}$ pigment type in organic solution; ${{\boldsymbol{K}}}_{{\boldsymbol{i}},{\boldsymbol{j}},{\rm{\Delta }}{\boldsymbol{\lambda }}}$ is the spectral displ.cement of the ${\boldsymbol{j}}{\rm{t}}{\rm{h}}$ absorption peak for the ${\boldsymbol{i}}{\rm{t}}{\rm{h}}$. pigment type *in vivo*; ${\boldsymbol{RA}}{{\boldsymbol{F}}}_{{\boldsymbol{i}},{\boldsymbol{j}},{\boldsymbol{lb}}}$ and ${\boldsymbol{RA}}{{\boldsymbol{F}}}_{{\boldsymbol{i}},{\boldsymbol{j}},{\boldsymbol{ub}}}$ stand for the ler and upper boundary of the ${\boldsymbol{j}}{\rm{th}}$ absorption peak for the *i*th pigment *in vivo* leaf. The main spectral characteristics of the pigment absorption coefficients are expressed by the metrics (${\boldsymbol{RA}}{{\boldsymbol{F}}}_{{\boldsymbol{i}},{\boldsymbol{j}},{\boldsymbol{lb}}}$ and ${\boldsymbol{RA}}{{\boldsymbol{F}}}_{{\boldsymbol{i}},{\boldsymbol{j}},{\boldsymbol{ub}}}$) in Table 1.

**Table 1:** Absorption peak characteristics determined from pigment absorption coefficients within PMP.

Specific absorption coefficient	Absorption peak	*K*_*i*,*j*,*v*_	*K*_*i*,*j*,*h*_(cm^2^/μg)	*K*_*i*,*j*,*w*_(nm)	*K*_*i*,*j*,*p*_(nm)	Δ*λ*_*i*,*j*_ (nm)	RAF (nm)
${K}_{Chla}$	j = 1	0.55	0.045	93	417	−15	400–464
	j = 2	0.72	0.003	103	590	10	—
	j = 3	0.98	0.011	108	626	8	—
	j = 4	0.44	0.032	23	680	16	668–692
${K}_{Chlb}$	j = 1	0.18	0.096	54	482	18	455–509
	j = 2	0.93	0.035	78	612	9	—
	j = 3	0.74	0.081	50	665	15	640–690
${K}_{C{\rm{ars}}}$	j = 1	0.53	0.049	46	520	50	497–543

Note that the symbol “—” stands for the negligible values in the RAFs because of the low absorbance values of these features. ${{K}}_{{\bf{i}},{\bf{j}},{\bf{v}}}$, ${{K}}_{{\bf{i}},{\bf{j}},{\bf{h}}}$ and ${{K}}_{{\bf{i}},{\bf{j}},{\bf{w}}}$ are the Gauss ratio, peak height and FWHM of the ${\boldsymbol{j}}{\rm{t}}{\rm{h}}$ absorption peak for the ${\boldsymbol{i}}{\rm{t}}{\rm{h}}$ pigment type *in vivo*, respectively; ${{K}}_{{\bf{i}},{\bf{j}},{\bf{p}}}$ is the peak position of the ${\boldsymbol{j}}{\rm{t}}{\rm{h}}$ absorption peak for the ${\boldsymbol{i}}{\rm{t}}{\rm{h}}$ pigment type; and ${{K}}_{{\bf{i}},{\bf{j}},{\rm{\Delta }}{\boldsymbol{\lambda }}}$ is the spectral displacement of the ${\boldsymbol{j}}{\rm{t}}{\rm{h}}$ absorption peak for the ${\boldsymbol{i}}{\rm{t}}{\rm{h}}$ pigment type *in vivo*.

**Figure 1:**
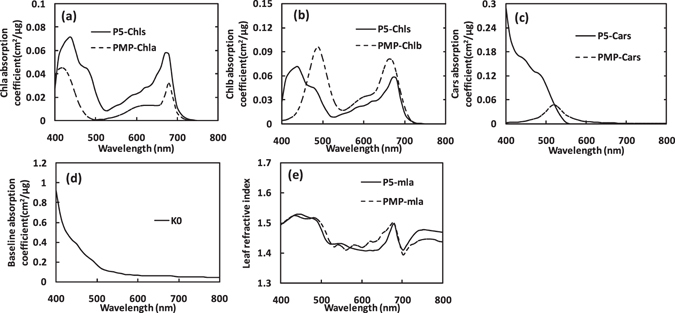
Spectral characteristics of the determined PROSPECT-5 (P5, solid line) and PROSPECT-MP (PMP, dotted line) parameters *in vivo* leaf. (**a**) show for Chla absorption coefficient (${{K}}_{{C}{h}{l}{a}}$); (**b**) for Chlb absorption coefficient (${{K}}_{{C}{h}{l}{b}}$); (**c**) for ${{K}}_{{C}{a}{r}{s}}$; (**d**) for leaf baseline absorption coefficient (${{K}}_{0}$); and (**e**) for leaf average refractive index (${\bar{{m}}}_{{l}{a}}$).

The spectra in Fig. 1 and data within Table 1 together allow us to describe the key characteristics of the pigment-specific and baseline absorption coefficients from PROSPECT-MP. For this parameter calibration, the characteristics of ${{\boldsymbol{K}}}_{{\boldsymbol{Chla}}}$ include: 1) two prominent absorption peaks (1^st^ and 4^th^) positioned at 417 and 680 nm; 2) spectral displacements of those peak positions relative to the corresponding absorption peaks in the organic solution (acetonitrile/methanol/dichloromethane; 60:20:20 v/v/v) are −15 and 16 nm, respectively; 3) the two main regions of absorption as defined by the RAFs are located at 400–464 and 668–692 nm, which are regions that have been used previously in empirical spectral indices for Chla^1, 3^.

The characteristics of ${{\boldsymbol{K}}}_{{\boldsymbol{Chlb}}}$ are: 1) two prominent absorption peaks (1^st^ and 3^rd^) at 482 and 665 nm with spectral displacements of 18 and 15 nm; 2) two RAFs located at 455–509 and 640–690 nm.

For ${{\boldsymbol{K}}}_{{\boldsymbol{Cars}}}$: 1) there is only one absorption peak which is located at 520 nm with a spectral displacement of 50 nm; 2) its absorption spectrum has a Gaussian shape and the RAF is 497–543 nm, which regions have been used for the previous spectral indices for Cars^18^. Attempts to separate specific absorption coefficients of individual pigments in the Cars group (Lu, An, Ze, Vi, Ne and β-Car) using the same method unfortunately failed. This could be due to the small distances between absorption peaks of individual pigments, which might mean that directional hemispherical reflectance (DHR) and transmittance (DHT) with higher spectral resolution are required for separating these pigments.

**Figure 2:**
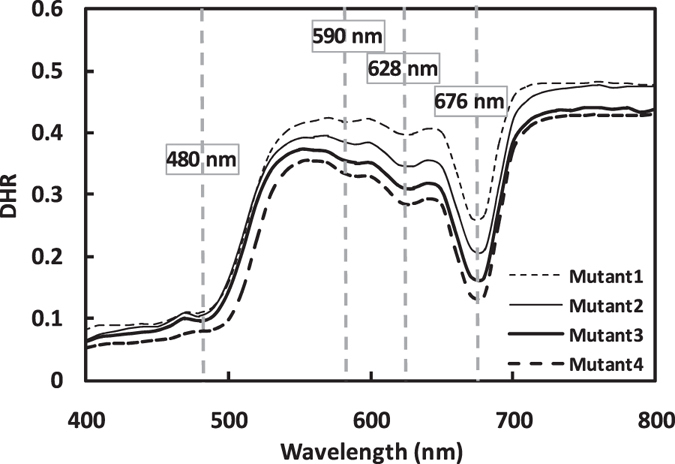
Leaf directional hemispherical reflectance (DHR) spectra which reveal the *in vivo* pigment absorption characteristics (Mutant1 = 11.864 μg/cm^2^; Mutant^2^ = 15.452 μg/cm^2^; Mutant3 = 20.521 μg/cm^2^ and Mutant4 = 27.944 μg/cm^2^).

The ${{\boldsymbol{K}}}_{0}$ spectrum shows a decreasing trend with wavelength (Fig. 2(d)), which is consistent with Jacquemoud and Baret^11^. Leaf average refractive index (${\bar{{\boldsymbol{m}}}}_{{\boldsymbol{la}}}$) shows similar general variations with wavelength change for both versions (Fig. 2(e)).

Figure 1(a and b) shows that the separated ${{\boldsymbol{K}}}_{{\boldsymbol{Chla}}}$ derived from PMP is reasonably consistent with the combined ${{\boldsymbol{K}}}_{{\boldsymbol{Chls}}}$ derived from P5, but this is not the case for ${{\boldsymbol{K}}}_{{\boldsymbol{Chlb}}}$. This could be the reason that Chla concentration is generally higher than Chlb in the LOPEX 93 dataset (Chla: Chlb ratio is around 3:1)^11^. Figure 2(c) shows that the form of the ${{\boldsymbol{K}}}_{{\boldsymbol{Cars}}}$ coefficient derived from PMP is consistent with absorption principles and differs substantially from the ${{\boldsymbol{K}}}_{{\boldsymbol{Cars}}}$ coefficient in P5.

As PROSPECT-MP uses the modified G-L function fitting, the determined pigment-specific absorption coefficients: 1) are all in accordance with the physical principles underpinning pigment absorption spectra^19^; 2) can directly account for peak position variations in environmental polarity between the organic solution and a leaf *in vivo* by using the spectral displacement parameter; 3) can also quantify the absorption characteristics of the corresponding pigment *in vivo* using the RAF parameter derived from FWHM.

#### Analysis of peak displacement within the absorption coefficients for PROSPECT-MP

**Figure 3:**
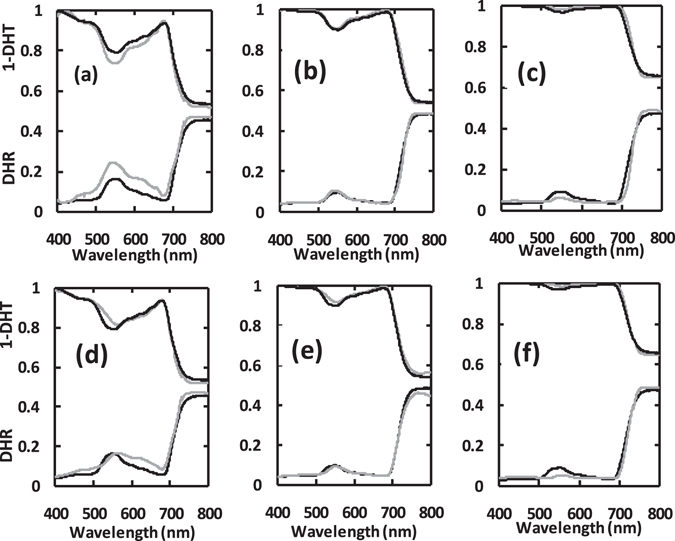
Comparison of measured (black) and simulated (grey) reflectance and transmittance for (**a**) low (Chla = 15.11 μg/cm^2^; Chlb = 4.25 μg/cm^2^; Cars = 5.94 μg/cm^2^), (**b**) medium (Chla = 47.86 μg/cm^2^; Chlb = 13.35 μg/cm^2^; Cars = 9.92 μg/cm^2^) and (**c**) high (Chla = 90.52 μg/cm^2^; Chlb = 29.34 μg/cm^2^; Cars = 27.41 μg/cm^2^) pigment concentration levels from P5; and (**d**) low, (**e**) medium and (**f**) high levels from PMP.

In order to evaluate the effectiveness of the spectral displacement parameter in accounting for shifts in the absorption peak positions *in vivo* compared with the organic solution, we compared the PROSPECT-MP pigment absorption coefficients (as described above) with reflectance spectra of leaves with particular biochemical compositions that revealed the absorption features of specific pigments *in vivo*. A set of leaves from a Chlb-deficient rice mutant (IG20) were provided by the College of Life Sciences, Zhejiang University, China^20^. In the 580–700 nm region, leaf reflectance is dominated by chlorophyll absorption, and in Fig. 3b, the reflectance spectra of the Chlb deficient leaves reveal the *in vivo* absorption characteristics of Chla. The absorption peaks for Chla are found at 590, 628 and 680 nm. In the 450–500 nm region, leaf absorption is dominated by Cars and Chlb. Thus, as Fig. 2 shows, the Chlb-deficient mutant leaves reveal the *in vivo* absorption peak for Cars (480 nm).

As noted earlier (Table 1) the absorption peaks of ***K***_*chla*_ (***λ***) (2^nd^, 3^rd^ and 4^th^ peaks) and Cars from PMP were observed at 590, 626, 680 and 520 nm, respectively, in which Chla and Chlb correspond closely with the *in vivo* absorption peaks (Fig. 2), but Cars performance is poor. This indicates that the process used to calibrate the Chla and Chlb absorption coefficients in PROSPECT-MP was effective. However, this analysis was not able to reveal all the *in vivo* absorption peaks for pigments (i.e. neither the Chlb peaks nor the 1^st^ Chla peak were determined). Nevertheless, this analysis did provide some evidence with which to evaluate the PROSPECT-MP absorption coefficients.

### Performance Evaluation

#### Spectral modelling performance

Figure 3 illustrates the performance of P5 and PMP in simulating directional hemispherical reflectance (DHR) and transmittance (DHT) for leaves at low, medium and high pigment levels. The performances for DHR and DHT are very similar at medium and high pigment levels. For the low pigment concentration, the simulations from P5 were worse than those at the other two concentration levels, with a tendency to underestimate reflectance and transmittance, which is consistent with the results from Feret *et al*.^11^. The main disparities are located in the 500–650 nm regions. Furthermore, for the low pigment concentration, PMP performs better than P5.

#### Spectral modelling evaluation

**Global performance evaluation of simulated leaf spectra**. The global performance assessment used averaged values across the whole spectrum (400–800 nm) for each evaluation metric (Root Mean Square Error, RMSE; BIAS; and Standard Error Corrected, SEC) from the measured and modeled spectrum of the 32 leaf samples (those not used for model calibration). For all model implementations, the results for DHR and DHT are encouraging in that the global RMSE values are less than 0.03, BIAS values are lower than ±0.01 and SEC values are similar to the global RMSE results (Table 2). These findings indicate that PROSPECT-MP can accurately compute leaf DHR and DHT using input data on Chla, Chlb and Cars concentrations, and its performance is similar to that of P5.

**Table 2:** Global performance evaluation of simulated leaf spectra from P5 and PMP (n = 32).

Spectrum type	Model implementation	RMSE	BIAS	SEC
DHR	**P5**	0.029	0.000	0.029
	**PMP**	0.025	0.009	0.022
DHT	**P5**	0.024	0.002	0.024
	**PMP**	0.022	0.008	0.019

**Figure 4:**
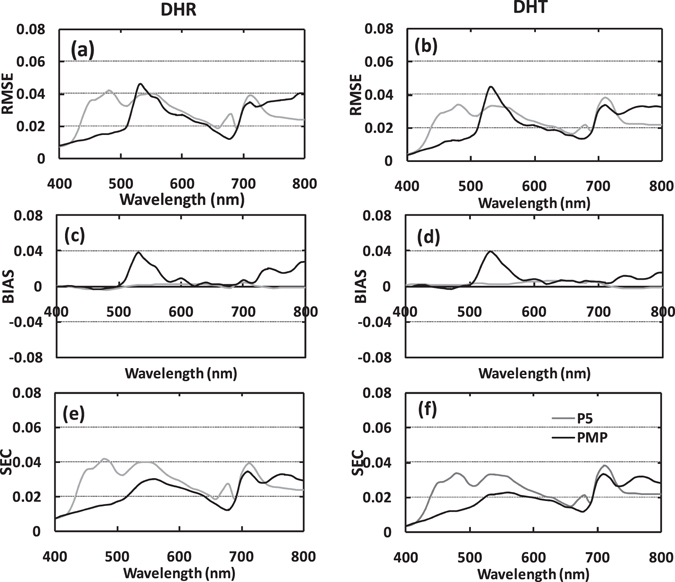
Simulated DHR (left column) and DHT (right column) spectra from P5 (grey line) and PMP (black line) (n = 32). (**a**) and (**b**) show RMSE values; (**c**) and (**d**) show BIAS; (**e**) and (**f**) show SEC.

**Local performance evaluation of simulated leaf spectra**. In contrast to the global evaluation, the local assessment is able to quantify errors in the leaf DHR and DHT simulations band-by-band. Figure 4 illustrates the results of the evaluation metrics from the two version implementations. RMSE values were variable across the whole spectrum. In the 460–510 nm regions where the absorption bands of Cars are located, PMP generated lower RMSE values for both leaf DHR and DHT modelling compared with P5. These results indicate that the PMP can better simulate leaf spectra by incorporating the contribution from Cars absorption. There are generally larger RMSE values in the 510–580 nm regions, where the anthocyanin absorption bands are located. Burger and Edwards^21^ reported that green leaves contain some determinable anthocyanin information, but the pigment was not considered in the LOPEX93 dataset. Thus, the incorporation of anthocyanin in the PROSPECT model will be required in subsequent studies. In the 600–700 nm region for Chls absorption, the RMSEs for DHR are lowest for PMP, then P5, which indicates that PROSPECT-MP can better simulate leaf DHR. In this region, the RMSEs for DHT are similar to DHR across the two version implementations. However, in the 730–800 nm region, the RMSE values for PMP are larger than P5 implementations. For the BIAS metric, the two version implementations perform similarly, except for PMP in the 510–580 nm region. For the SEC metric, the results for the two inversion implementations are similar to those for RMSE. These results demonstrate that PROSPECT-MP can successfully simulate leaf spectra in a similar fashion to PROSPECT-5, but PROSPECT-MP appears to perform better in the Cars absorption bands.

#### Pigment concentration retrieval performance

The capacity of the PROSPECT model to retrieve the concentrations of pigments from spectra of fresh leaves depends on the types of pigment absorption coefficients used in the model. Hence, the P5 and PMP implementations tested here differ in their capacity to retrieve different pigments: P5 can only directly retrieve Chls and Cars; while PMP can retrieve Chla, Chlb and Cars. As Chls in a leaf is usually the sum of Chla and Chlb^22^, we can derive Chls from PMP, to facilitate the comparison.

**Figure 5:**
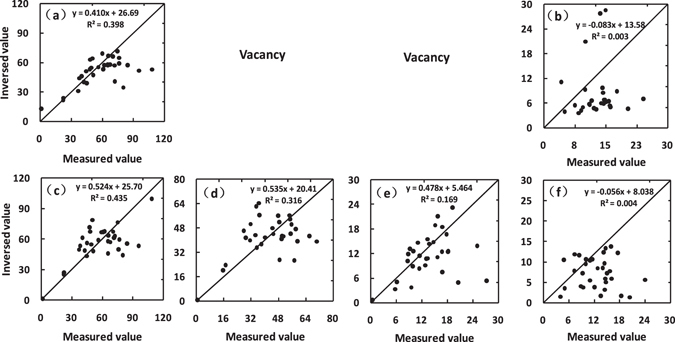
Comparison between measured and retrieved pigment concentrations (µg/cm^2^, n = 32). (**a**) and (**c**) are for Chls concentration; (**d**) is Chla; (**e**) is Chlb; and (**b**) and (**f**) are Cars; Retrievals (**a**) and (**b**) are from inversions of P5; (**c**), (**d**), (**e**) and (**f**) from PMP.

Figure 5 Illustrates the relationships between measured pigment concentrations and the corresponding values retrieved from the measured spectrum of the 32 leaf samples (those not used for model calibration) by model inversion. Vacant plots denote that P5 implementation is incapable of retrieving the specific pigment. The results show that the performance of PMP enables the retrieval of Chls, Chla, Chlb and Cars concentration from leaf spectra, but P5 only does for Chls and Cars.

#### Pigment concentration retrieval evaluation

The evaluation metrics (RMSE, BIAS, SEC and Coefficient Variability (CV)) for the retrieved concentrations are shown in Table 3. For Chls retrieval, the performance of the two version implementations is similar, but with PMP performing better than P5 in terms of RMSE and BIAS. For Cars retrieval, PMP model implementations substantially outperformed P5. Thus, the results demonstrate that for Chls concentration retrievals, PROSPECT-MP and PROSPECT-5 have a similar performance, while PROSPECT-MP produces a reliable estimate of Chla and Chlb concentrations and a more accurate estimate of Cars than PROSPECT-5.

**Table 3:** The validation of pigment concentration retrievals from *in vivo* leaf spectra by P5 and PMP (n = 32).

Performance types	P5		PMP			
Pigment types	Chls	Cars	Chls	Chla	Chlb	Cars
RMSE (μg/cm^2^)	18.25	16.11	16.51	14.87	4.65	8.93
BIAS (μg/cm^2^)	−7.45	−0.43	−1.80	0.02	−1.82	−3.23
SEC (μg/cm^2^)	16.60	16.11	16.67	13.11	6.49	5.74
CV (%)	28.67	128.22	28.79	33.32	35.03	35.69

Although PROSPECT-MP can provide a reliable retrieval capability of Chls and Cars concentration comparing to PROSPECT-5, and can also retrieve Chla and Chlb concentrations, these performances of pigment retrieval from PMP are still weak based on the R^2^ values and regression equations in Fig. 5, especially Chlb and Cars. The reasons will be given as follows. When the G-L function fitting method is used to separate the mixed absorption spectra with band overlapping features, two requirements should be met: 1) All material responded on spectra must be considered^14^. Plant leaves commonly contain the determinable Ants information^21^, but in the LOPEX93 dataset, Anthocyanins (Ants) was not considered. Due to band overlapping features in the absorption spectra of Ants and Chlb and Cars^3, 4^, the case of unconsidering Ants in PMP could produce the distorted calibration of pigment absorption coefficients, leading to weak performance of pigment retrievals, especially Chlb and Cars. 2) The measurements of all material should be accurate enough. In the LOPEX93 dataset^23^, the spectrophotometric method for Cars measurement might forget its component pigments (lutein, neoxanthin, etc.), which could lead to the measurements of Cars not accurate enough^24^. The inaccurate measurements of Cars could firstly produce the over-fitting of pigment absorption coefficients using a minimum distance fitting method^25^, and then lead to the distorted absorption coefficients of Chla, Chlb and Cars, ultimately resulting in weak performances of these pigment retrievals.

Based on the analysis above, there are two probable causes for the poor performances of pigment retrievals in PMP: 1) Anthocyanins (Ants) was not considered in the LOPEX93 dataset. 2) The measurement of Cars was not accurate enough. Thus, a consideration of Ants information and an improvement of the measurement of individual photosynthetic pigment concentrations are needed in the future studies for the more accurate retrievals of plant pigments from leaf spectra.

## Conclusions

This paper demonstrates that the extended version of PROSPECT (PROSPECT-MP) proposed in this study can reliably simulate leaf hemispherical reflectance and transmittance in the 400–800 nm region, and it can retrieve accurately, multiple photosynthetic pigment concentrations comparing to PROSPECT-5 from spectra of fresh leaves by model inversion. The modified G-L function is employed to create a new function for the overall leaf absorption coefficient, which can limit the masking phenomenon between pigments. Consequently PROSPECT-MP is parameterized using pigment absorption coefficients (***K***_***chla***_ (***λ***), ***K***_***chlb***_ (***λ***), and ***K***_***cars***_ (***λ***)). The determined pigment absorption coefficients possess three key features: 1) they are consistent with the physical principles of pigment absorption spectra; 2) they account for the spectral displacement of absorption peaks within media of different polarities; 3) they quantify the main absorption characteristics of each pigment with the RAF parameter.

In order to test the effectiveness of these developments in the treatment of leaf optics within PROSPECT-MP, the LOPEX93 dataset was used to evaluate the ability of PROSPECT-MP to simulate leaf DHR and DHT spectra and retrieve pigment concentrations from measured fresh leaf spectra by model inversion. To provide some context, the performance of PROSPECT-MP was compared with that of PROSPECT-5. The results were encouraging in that: 1) PROSPECT-MP was able to simulate accurately, *in vivo* leaf DHR and DHT spectra; 2) PROSPECT-MP can be used to retrieve leaf Chls and Cars with similar accuracies to PROSPECT-5; and, 3) PROSPECT-MP provides an additional capability for retrieving individual Chla and Chlb concentrations.

Our ongoing work is now focusing on improving the description within PROSPECT-MP of the optical properties of leaf photo-protective pigment (anthocyanins). Future developments of PROSPECT-MP would improve the robustness and transferability in the capabilities for retrieval of multiple pigment concentrations, and a synthesis of PROSPECT-MP with a canopy RT model will offer new opportunities for improving the estimates of pigment variations across larger spatial scales. PROSPECT-MP will provide a framework for future developments in the modelling of leaf optical properties and for the hyperspectral remote sensing of vegetation biochemistry.

## Data and Method

### Data

The LOPEX93 dataset^23^ and the absorption spectrum of pure pigments in an organic solution were used for calibrating and evaluating the performance of PROSPECT-MP. The LOPEX93 dataset has been used previously for the calibration and validation of leaf and canopy RT models^26–28^ and incorporates a wide range of biochemical concentrations and plant species. Therefore, in the present study, PROSPECT-MP was calibrated and compared with the PROSPECT-5 model using this dataset. To achieve this, we selected all 64 single-fresh leaf samples from the LOPEX93 dataset for which DHR and DHT were available along with measures of Chla, Chlb, Cars content^11^. In order to determine the numbers of absorption peaks and their positions for individual pigments, absorption spectra were obtained for pure pigments (Chla, Chlb, β-Car, Vi, An, Ze, Ne, and Lu) in a mixed organic solution using a Shimadzu UV-VIS detector^29^. The pigments and the organic solution were of chromatographic purity and purchased from the Sigma-Aldrich Co. LLC in January 2013.

### Developing PROSPECT-MP method

#### Principles of PROSPECT-MP

**Description of leaf absorption coefficient function**. In fresh leaves, the presence of Chla, Chlb and Cars can influence the observed reflectance and transmittance spectra^3, 4^. However, leaf absorption coefficient (${\boldsymbol{k}}$) of PROSPECT-5 incorporates Chls and Cars without separating Chla and Chlb. Therefore, PROSPECT-5 cannot simulate the influence of Chla and Chlb on leaf spectra and cannot retrieve the Chla and Chlb from spectra by model inversion. To overcome the problem, we extended the pigment absorption function to incorporate Chla, Chlb and Cars leaf absorption coefficients, and these were described based on previous reports^11, 12, 30^.

2 $$k(\lambda )=\frac{{K}_{Chla}(\lambda ){C}_{Chla}+{K}_{Chlb}(\lambda ){C}_{Chlb}+{K}_{Cars}(\lambda ){C}_{Cars}}{N}+{K}_{0}(\lambda )$$

where ${\boldsymbol{N}}$ stand leaf structure index.

**Characterization of pigment absorption coefficients** ***in vivo*** **leaf using modified Gauss-Lorentz function**. Although Chla, Chlb and Cars have selective absorption characteristics, there is considerable overlap in their absorption spectra^3, 31^ and this can make the masking phenomenon to block the simultaneous separation of these individual pigment absorption coefficients in equation 2. In order to limit masking in the separation of these pigment absorption features, our approach was to employ a modified G-L function fitting method which uniformly describes the peaks of multiple individual pigment absorption coefficients *in vivo* leaf, as given in the expression below.

3 $${K}_{i,j}(\lambda )={K}_{i,j,v}\cdot {K}_{i,j,h}\cdot {e}^{-4ln2\cdot {(\frac{{A}_{i,j,p}+{K}_{i,j,{\rm{\Delta }}\lambda }-\lambda }{{K}_{i,j,w}})}^{2}}+(1-{K}_{i,j,v})\frac{{K}_{i,j,h}}{1+4{({A}_{i,j,p}+{K}_{i,j,{\rm{\Delta }}\lambda }-\lambda )}^{2}{{K}_{i,j,w}}^{-2}}$$

where$\,{{\boldsymbol{K}}}_{{\boldsymbol{i}},{\boldsymbol{j}}}$ represents the ${\boldsymbol{j}}{\rm{t}}{\rm{h}}$ peak function within the absorption coefficient for the ${\boldsymbol{i}}{\rm{t}}{\rm{h}}$ pigment type; The factor ${{\boldsymbol{K}}}_{{\boldsymbol{i}},{\boldsymbol{j}},{\rm{\Delta }}{\boldsymbol{\lambda }}}$ is introduced to account for the shifts in absorption peak positions of specific pigments *in vivo* compared with the organic solution extract^4, 32^, and they when linked to the corresponding ${{\boldsymbol{A}}}_{{\boldsymbol{i}},{\boldsymbol{j}},{\boldsymbol{p}}}$, can help the modified G-L function to index and characterize the corresponding potential absorption region of ${{\boldsymbol{K}}}_{{\boldsymbol{i}},{\boldsymbol{j}}}$. This treatment can limit the masking phenomenon in the separation of multiple pigment absorption coefficients.

As the pigment absorption feature comes from the sum of absorption features of each peak, the pigment-specific absorption coefficients can be expressed as:

4 $${K}_{i}(\lambda )=\sum _{j=1}^{j}{K}_{i,j}(\lambda ).\,$$

These pigment-specific absorption coefficients then allow us to solve for *K* in equation 2.

For the G-L function, the determination of the pigment absorption number and positions of peaks is the key issue for the parameterization of absorption spectra, which will be given as follows.

**Determination of pigment absorption peak number and position for the modified G-L function**. The observed number of absorption peaks for photosensitive material in a weaker polarity medium can be regarded as the potential maximum number in a stronger polarity medium^33^. For fresh leaves, the water content is commonly over 50%^11^. Therefore, leaves can be regarded as essentially a water-based medium. Cotton *et al*.^34^ reported the polarity order of different media as: water > acetonitrile > methanol > dichloromethane. Hence, the polarity of leaf pigments *in vivo* is greater than that in the mixed organic solution (acetonitrile/methanol/dichloromethane; 60:20:20 v/v/v). As a result, the observed number of absorption peaks for pure pigments in the mixed organic solution can be regarded as the potential number of absorption peaks of the corresponding pigments *in vivo*.

**Figure 6:**
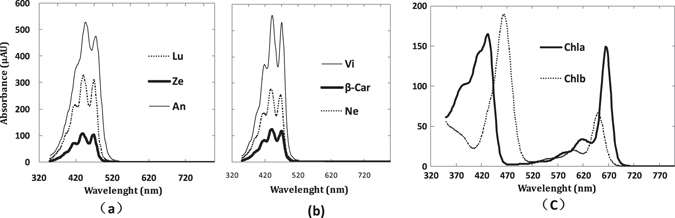
Absorption spectra of pure pigments in acetonitrile/methanol/dichloromethane (60:20:20 v/v/v). The concentrations of Lu (Lutein), An (Antheraxanthin), Ze (Zeaxanthin) in (**a**) and Ne (Neoxanthin), Vi (Violaxanthin), β-Car (β-carotene) in (**b**) were all 0.2 mg/ml; Chla and Chlb in (**c**) were 0.01 mg/ml.

The absorption spectra of pure pigments in mixed organic solution are shown in Fig. 6. It can be seen that the positions of the three absorption peaks are adjacent within Cars group (Fig. 6(a) and (b)). Table 4 demonstrates that the largest distance between absorption peak positions within the Cars group did not exceed 8 nm. Hence, we merged all of the individual peak positions from Lu, An, Ze, Vi, Ne and β-Car into a single set of peak positions to represent the Cars and the averaged value of these peaks are for the peak positions for the Cars. Therefore, the determinable leaf pigment groups are Chla, Chlb and Cars. The number and positions of absorption peaks were obtained by calculating first and second derivatives of the absorption spectra and the results are shown in Table 4. Given the above argument about differences in environmental polarity^35^, Table 4 shows the potential maximum number of peaks within ***A***_***Chla***_, ***A***_***ChlB***_, and ***A***_***Cars***_ *in vivo*.

**Table 4:** The number of absorption peaks and their wavelength positions in the 400–800 nm region, for pure pigments in a mixed organic solution.

Absorption peak no.	${{\boldsymbol{A}}}_{{\boldsymbol{Chla}},j,{\boldsymbol{p}}}\,$(nm)	${{\boldsymbol{A}}}_{{\boldsymbol{Chlb}},j,{\boldsymbol{p}}}\,$(nm)	${{\boldsymbol{A}}}_{{\boldsymbol{Cars}},j,{\boldsymbol{p}}}\,$(nm)						
	Chla	Chlb	Cars	Lu	An	Ze	Vi	Ne	β-Car
j = 1	432	458	418	422	422	416	418	414	416
j = 2	580	602	443	450	448	440	442	438	440
j = 3	618	650	470	476	474	468	470	466	468
j = 4	664	—	—	—	—	—	—	—	—

Note that the absorption peak positions for Cars are based on the average positions of Lu, An, Ze, Vi, Ne and β-Car which were spectrally adjacent.

**Extension of PROSPECT to PROSPECT-MP for multiple pigment retrieval**. In the PROSPECT model, leaf DHR (${{\boldsymbol{R}}}_{{\boldsymbol{N}}}$) and DHT (${{\boldsymbol{T}}}_{{\boldsymbol{N}}}$) are given^11^ by:

5 $${R}_{N}={R}_{1{\rm{st}}}+\frac{{T}_{1{\rm{st}}}{R}_{N-1}T}{1-{R}_{N-1}R};\,{T}_{N}=\frac{{T}_{1{\rm{st}}}{T}_{N-1}}{1-{R}_{N-1}R}$$

where ${{\boldsymbol{R}}}_{1{\bf{s}}{\bf{t}}}$ and ${{\boldsymbol{T}}}_{1{\bf{s}}{\bf{t}}}$, ${\boldsymbol{R}}$ and ${\boldsymbol{T}}$, **and** ${{\boldsymbol{R}}}_{{\boldsymbol{N}}-1}$ and $\,{{\boldsymbol{T}}}_{{\boldsymbol{N}}-1}$ are the DHR and DHT of the first layer, the internal elementary layer and the internal *N*-1elementaayers. They can be parameterized by parameters N and ${\bar{{\boldsymbol{m}}}}_{{\boldsymbol{la}}}$ and ${\boldsymbol{\tau }}$. (leaf transmission coefficient). The paramter ${\boldsymbol{\tau }}$. is related to${\boldsymbol{k}}({\boldsymbol{\lambda }})$ through the following equation^36^:

6 $$\tau =(1-k(\lambda )){e}^{-k(\lambda )}+k{(\lambda )}^{2}{\int }_{\,k(\lambda )}^{\infty }{x}^{-1}{e}^{-x}dx$$

With ${\boldsymbol{k}}$ derived as in equation 2, incorporating the absorption coefficients of multiple individual photosynthetic pigments *in vivo*, we use the improved $k$ in equation 2 to replace $k$ of PROSPECT model in equation 6 to extend the model at 400–800 nm to a leaf optical model (PROSPECT-MP) for the simultaneous retrieval of multiple individual photosynthetic pigments (Chla, Chlb and Cars).

In addition, the methods of calibration and evaluation for PROSPECT-MP and PROSPECT-5 in the LOPEX93 dataset were shown in the Note S1 and S2 of ‘Supplementary Information’.

## Electronic supplementary material

Supplementary Information

## References

[1] Sims DA, Gamon JA (2002). Relationships between leaf pigment content and spectral reflectance across a wide range of species, leaf structures and developmental stages. Remote Sens. Environ..

[2] Jensen, J. R. Remote sensing of the environment: An earth resource perspective 2nd ed (ed. Jensen, J.) 355-356 (Prentice-Hall 2002).

[3] Blackburn GA (2007). Hyperspectral remote sensing of plant pigments. J. Exp. Bot..

[4] Ustin SL (2009). Retrieval of foliar information about plant pigment systems from high resolution spectroscopy. Remote Sens. Environ..

[5] Rahimzadeh-Bajgiran P, Munehiro M, Omasa K (2012). Relationships between the photochemical reflectance index (PRI) and chlorophyll fluorescence parameters and plant pigment indices at different leaf growth stages. Photosynth. Res..

[6] Raven, P. H., Evert, R. F. & Eichhorn, S. E. Biology of Plants 7th ed (7th ed.) 119–127 (W. H. Freeman and Co. 2005).

[7] Ishikita H, Saenger W, Biesiadka J (2006). How photosynthetic reaction centers control oxidation power in chlorophyll pairs P680, P700, and P870. P Nati. Acad. Sci. USA, 2006.

[8] Young AJ (1991). The photoprotective role of carotenoids in higher plants. Physio. Plantarum.

[9] Kura-Hotta M, Satoh K, Katoh S (1987). Relationship between photosynthesis and chlorophyll content during leaf senescence of rice seedlings. Plant Cell Physiol..

[10] Kitajima K, Hogan KP (2003). Increases of chlorophyll a/b ratios during acclimation of tropical woody seedlings to nitrogen limitation and high light. Plant Cell Environ..

[11] Feret JB (2008). PROSPECT-4 and 5: Advances in the leaf optical properties model separating photosynthetic pigments. Remote Sens. Environ..

[12] Jacquemoud S, Baret F (1990). PROSPECT: A model of leaf optical properties spectra. Remote Sens. Environ..

[13] Jacquemoud S (2009). PROSPECT+ SAIL models: A review of use for vegetation characterization. Remote Sens. Environ..

[14] Namjou K (1998). Sensitive absorption spectroscopy with a room-temperature distributed-feedback quantum-cascade laser. Opt. Lett..

[15] Vanhamme L, Sundin T, Hecke PV, Huffel SV (2001). MR spectroscopy quantitation: a review of time-domain methods. NMR Biomed..

[16] James TM (2013). Automated quantitative spectroscopic analysis combining background subtraction, cosmic ray removal, and peak fitting. Appl. Spectros..

[17] Brown AJ (2006). Spectral curve fitting for automatic hyperspectral data analysis. IEEE T Geosci. Remote..

[18] Zhao Y, Li X, Yu K, Cheng F, He Y (2016). Hyperspectral Imaging for Determining Pigment Contents in Cucumber Leaves in Response to Angular Leaf Spot Disease. Sci. Rep..

[19] Siano DB, Metzler DE (1969). Band shapes of the electronic spectra of complex molecules. J. Chem. Phys..

[20] Zhang J, Huang W, Zhou Q (2014). Reflectance Variation within the In-Chlorophyll Centre Waveband for Robust Retrieval of Leaf Chlorophyll Content. Plos One.

[21] Burger J, Edwards GE (1996). Photosynthetic Efficiency, and Photodamage by UV and Visible Radiation, in Red versus Green Leaf Coleus Varieties. Plant Cell Physiol..

[22] Gitelson AA, Gritz Y, Merzlyak MN (2003). Relationships between leaf chlorophyll content and spectral reflectance and algorithms for non-destructive chlorophyll assessment in higher plant leaves. J Plant Physiol..

[23] Hosgood, B. *et al*. Leaf optical properties experiment 93 (LOPEX93). European Commission (1995).

[24] Šesták, Z. Chlorophylls and Carotenoids during Leaf Ontogeny: Photosynthesis during leaf development 1st ed (1st ed.) 76–106 (Springer Netherlands 1985).

[25] Cawley GC, Talbot NLC (2010). On Over-fitting in Model Selection and Subsequent Selection Bias in Performance Evaluation. J Mach. Learn, Res..

[26] Jacquemoud S, Bacour C, Poilvé H, Frangi JP (2000). Comparison of Four Radiative Transfer Models to Simulate Plant Canopies Reflectance: Direct and Inverse Mode. Remote Sens. Environ..

[27] le Maire G, François C, Dufrêne E (2004). Towards universal broad leaf chlorophyll indices using PROSPECT simulated database and hyperspectral reflectance measurements. Remote Sens. Environ..

[28] Zou X, Mõttus M (2015). Retrieving crop leaf tilt angle from imaging spectroscopy data. Agr. Forest Meteorol..

[29] De Las Rivas J, Abadía A, Abadía J (1989). A new reversed phase-HPLC method resolving all major higher plant photosynthetic pigments. Plant Physiol..

[30] Jacquemoud S (1996). Estimating leaf biochemistry using the PROSPECT leaf optical properties model. Remote Sens. Environ..

[31] Merzlyak MN, Gitelson AA, Chivkunova OB, Rakitin VY (1999). Non-destructive optical detection of pigment changes during leaf senescence and fruit ripening. Physiol. Plantarum..

[32] Riddle C, Smith TE (1977). The application of a ‘curve-fitting computer program’ to compensate for inter-element effects in X-ray fluorescence analysis of rock samples. The determination of vanadium in geological standards. X-Ray Spectrom..

[33] Ebbing, D. D. & Wrighton, M. S. General Chemistry 2nd ed (ed: Ebbing, D.) 327 (Houghton Mifflin Company 1996).

[34] Cotton, F. A. & Wilkinson, G. Chromium. In: Advanced inorganic chemistry: a comprehensive text 4th ed (ed: Cotton F.) 719–736 (Wiley 1980).

[35] Peters RD, Noble SD (2014). Spectrographic measurement of plant pigments from 300 to 800 nm. Remote Sens. Environ..

[36] Allen WA, Gausman HW, Richardson AJ, Thomas JR (1969). Interaction of isotropic light with a compact plant leaf. Jopt. Soc. Am. B..

