# Supplementary material for: An extended PROSPECT: Advance in the leaf optical properties model separating total chlorophylls into chlorophyll a and b
Source: Sci Rep. 2017 Jul 25;7:6429. doi: 10.1038/s41598-017-06694-y (PMC5526878; doi:10.1038/s41598-017-06694-y)
Supplement: Supplementary file 1 — Supplementary Information [file 41598_2017_6694_MOESM1_ESM.pdf]

Supplementary Information

Article title: An extended PROSPECT：Advance in the leaf optical properties model separating total chlorophylls into chlorophyll a and b

Authors: Yao Zhang, Jingfeng Huang, Fumin Wang, George Alan Blackburn, Hankui K Zhang, Xiuzhen Wang, Chuanwen Wei, Kangyu Zhang, Chen Wei.

The following Supporting Information is available for this article:

NOTE S1 Calibration of PROSPECT-MP parameters.
NOTE S2 Evaluating the performance of PROSPECT-MP.

NOTE S1 Calibration of PROSPECT-MP parameters

In this study, parameter calibration using the empirical dataset was performed for three sub-steps: determination of leaf structure index; determination of leaf baseline absorption coefficient; determination of pigment-specific absorption coefficients and leaf average refractive index. Here, the determination of leaf structure index based on the reported method by Feret *et al.*[1] is not shown in this paper.

Determination of $\boldsymbol{K_0}$

The albino leaf was used to determine $\boldsymbol{K_0}$. As there are trace pigments in albino leaves, the albino leaf absorption function ($\boldsymbol{k_{albino}}$) is expressed as:

$$\boldsymbol{k_{albino}}(\boldsymbol{\lambda}) = \frac{\sum \boldsymbol{C_{i,0}}\, \boldsymbol{K_0}(\boldsymbol{\lambda})/\boldsymbol{n_0}}{\boldsymbol{N_0}} + \boldsymbol{K_0}(\boldsymbol{\lambda}) \quad (\boldsymbol{S1})$$

where $\boldsymbol{N_0}$ is the structure index of the albino leaf; $\boldsymbol{C_{i,0}}$ is the concentration of the ***ith*** pigment ($\boldsymbol{i}$ can be Chla, Chlb, Cars or Ants) within the albino leaf $\boldsymbol{0}$; $\boldsymbol{n_0}$ represents the number of pigment types. The optical properties and pigment concentration of albino leaves from Sugar maple were measured using the methods of Hosgood *et al.*[2] to obtain some auxiliary data to support the study.

$\boldsymbol{K_0}$ was derived from the albino leaf data using Minimum Distance Fitting of Spectra[3] with a Least Squares Optimization[4] (MDFS&LSO) which minimizes the merit function ($\boldsymbol{\chi}$):

$$\boldsymbol{\chi}(\boldsymbol{K_0}(\boldsymbol{\lambda})) = \sum_{\boldsymbol{\lambda=400}}^{\boldsymbol{800}} \left(\boldsymbol{R_{mea,0}}(\boldsymbol{\lambda}) - \boldsymbol{R_{mod,0}}(\boldsymbol{\lambda})\right)^{\boldsymbol{2}} + \left(\boldsymbol{T_{mea,0}}(\boldsymbol{\lambda}) - \boldsymbol{T_{mod,0}}(\boldsymbol{\lambda})\right)^{\boldsymbol{2}} \ (\boldsymbol{S2})$$

where $\boldsymbol{R_{mea,0}}$ and $\boldsymbol{T_{mea,0}}$, $\boldsymbol{R_{mod,0}}$ and $\boldsymbol{T_{mod,0}}$ are the measured and simulated reflectance and transmittance of the albino leaf, respectively.

Determination of $\boldsymbol{K_i}$ *and* $\boldsymbol{\overline{m}_{la}}$

In the LOPEX93 dataset, the measured leaf pigments are Chla, Chlb and Cars. Therefore, this

permitted the calibration of the pigment-specific absorption coefficients $\boldsymbol{K_{Chla}}$, $\boldsymbol{K_{Chlb}}$ and $\boldsymbol{K_{Cars}}$ and $\boldsymbol{\bar{m}_{la}}$ within PROSPECT-MP. In order to provide some context for performance evaluation, PROSPECT-MP was compared with the PROSPECT-5 at 400-800 nm. To enable a direct comparison, the data for the calibration of PROSPECT-MP was also used to calibrate $\boldsymbol{K_{Chls}}$, $\boldsymbol{K_{Cars}}$ and $\boldsymbol{\bar{m}_{la}}$ within PROSPECT-5. Table S1 shows the details of various parameter calibrations that were implemented, showing the number of leaf samples used, input variables, algorithm employed and output variables. Having selected 32 leaf samples from the LOPEX93 dataset for model calibration, the remaining 32 leaf samples in the dataset were subsequently used in the corresponding model validation.

Table S1. Details of the parameter calibrations of PROSPECT-MP (PMP) and PROSPECT-5 (P5).

Calibration Type	No. leaf samples	Input variables	Algorithm	Output variables	Description
P5	32	$C_{mea,i}$, R_{mea}, T_{mea} and N	MDFS&LSO at each wavelength	K_i, $\bar{m}_{la}$	i can be Chls or Cars.
PMP	32	$C_{mea,i}$, R_{mea}, T_{mea}, K_0, $A_{ij,p}$ and N	MDFS&LSO for all wavelengths	K_i, $\bar{m}_{la}$	i can be Chla, Chlb or Cars.

Note that $\boldsymbol{R_{mea}}$, $\boldsymbol{T_{mea}}$, $\boldsymbol{C_{mea,i}}$ stand for the measured DHR, DHT and pigment concentration; $\boldsymbol{A_{i,j,p}}$ is the position of pigment absorption peak in the organic solution from Table 4.

NOTE S2 Evaluating the performance of PROSPECT-MP

To evaluate the performance of PROSPECT-MP for modelling leaf spectra and retrieving pigment concentrations by model inversion, we designed the different comparisons based on various implementations of the PROSPECT-MP and PROSPECT-5 using LOPEX 93 dataset for: (i) forward modelling of leaf DHR and DHT; and (ii) retrieval of pigment concentrations by model inversion. Table S3 shows the different implementations of different PROSPECT model versions. Details are also provided in Table S3 on the number of leaf samples used, the input and output variables, and the algorithm employed.

Table S3. Implementations of PROSPECT-MP (PMP) and PROSPECT-5 (P5) for spectral modelling and pigment retrieval by model inversion. $\mathbf{R_{mea}}$, $\mathbf{T_{mea}}$ and $\boldsymbol{C_{mea,i}}$ stand for the measured leaf DHR, DHT and pigment concentration and $\mathbf{R_{mod}}$, $\mathbf{T_{mod}}$ and $\boldsymbol{C_{inv,i}}$ for the modeled or retrieved values.

model version	Implementation	Leaf samples	Input variables	Algorithm	Output variable	Description
P5	Forward spectral modelling	32^A	$\boldsymbol{C_{mea,i}}$, K_i, $\bar{m}_{la}$ and N	Direct computing for each leaf sample	R_{mod}, T_{mod}	$\boldsymbol{i}$ can be Chls or Cars, $\boldsymbol{K_i}$, $\boldsymbol{\bar{m}_{la}}$ from P5.
P5	Inversion for pigment retrieval	32^A	R_{mea}, T_{mea}, K_i, $\bar{m}_{la}$ and N	MDFS&LSO for each leaf sample	$\boldsymbol{C_{inv,i}}$	$\boldsymbol{i}$ can be Chls or Cars, $\boldsymbol{K_i}$, $\boldsymbol{\bar{m}_{la}}$ from P5.
PMP	Forward spectral modelling	32^A	$\boldsymbol{C_{mea,i}}$, K_i,K_0, $\bar{m}_{la}$, N	Direct computing for each leaf sample	R_{mod}, T_{mod}	$\boldsymbol{i}$ can be Chla, Chlb or Cars, $\boldsymbol{K_i}$, $\boldsymbol{\bar{m}_{la}}$ from PMP .
PMP	Inversion for pigment retrieval	32^A	R_{mea}, T_{mea}, K_i,K_0, $\bar{m}_{la}$, N	MDFS&LSO for each leaf sample	$\boldsymbol{C_{inv,i}}$	$\boldsymbol{i}$ can be Chla, Chlb or Cars, $\boldsymbol{K_i}$, $\boldsymbol{\bar{m}_{la}}$ from PMP .

Note that the superscript denotes A is the number of leaf samples used for P5 or PMP validation from the LOPEX93 dataset.

References

1. Feret, J.B., François, C., Asner, G.P., Gitelson, A.A., Martin, R.E., Bidel, L.P.R., Ustin, S.L., le Maire, G., & Jacquemoud, S. PROSPECT-4 and 5: Advances in the leaf optical properties model separating photosynthetic pigments. *Remote Sens. Environ.* 112, 3030-3043 (2008).

2. Hosgood, B., Jacquemoud, S., Andreoli, G., Verdebout, J., Pedrini, G., & Schmuck, G. Leaf optical properties experiment 93 (LOPEX93). *European Commission* (1995)

3. Jacquemoud, S., & Baret, F. PROSPECT: A model of leaf optical properties spectra. *Remote Sens. Environ.* 34, 75-91 (1990).

4. Arai K., Moriyama M., Terayama Y., & Ueda Y. Adaptive least squares method for estimation of partial cloud coverage within a pixel. *Int. J. Remote Sen.* 16, 2197-2206 (1995).
